# Intra-abdominal ectopic breast tissue in male patient presenting as a fibroadenoma: a case report

**DOI:** 10.1097/MS9.0000000000000332

**Published:** 2023-03-25

**Authors:** Zekrullah Baset, Yaser Arafat, Jawad Amini, Fatema Rezaie, Nooria Mohammady, Sayed H. Mousavi

**Affiliations:** a Departments of Histopathology and Cytopathology, Human Medical Laboratories; b Kocasinan Mahallesi, Sivas bulvari, Kocasinan, Kayseri, Turkiye; c Kabul University of Medical Sciences; d Faculty of Medicine; e Medical Research Center, Kateb University, Kabul, Afghanistan

**Keywords:** Afghanistan, ectopic breast, fibroadenoma, histopathologic, intra-abdomen

## Abstract

Fibroadenoma (FA) in ectopic male breast tissue is extremely rare. The ectopic breast tissue (EBT) is mostly appearing along the milk line; however, uncommon locations are also seen, such as the present case.

**Case Presentation:**

The authors report a 19-year-old male presenting with intestinal obstruction. The patient underwent laparoscopic surgery, and an excisional biopsy of the lesion was done. The histopathologic result confirms FA arising from EBT. This case is reported for its rarity. It indicates that FA should always be considered when there is an intra-abdominal suspicious mass.

**Discussion:**

EBT presenting as FA is also reported in the face, posterior neck, chest, middle back, buttocks, vulvar, and thighs. In this case, the authors present a EBT presented as a FA in the intra-abdomen of a young male patient, causing intestinal obstruction. FA in the male breast is rare; however, benign breast parenchyma showing FA in the intra-abdomen of a male patient is extremely rare.

**Conclusion:**

When a tumor is palpated in the milk line, the existence of FA should be taken into consideration. FA of the male EBT in the intra-abdomen is extremely rare. However, a close follow-up of the patient is strongly recommended, as the carcinoma arising from FA has a very poor prognosis.

## Introduction

Highlights

- Ectopic breast tissue (EBT) presenting as fibroadenoma (FA) is a very rare entity; it may be milk like.
- This case presents EBT presenting as FA in an adult male, which is extremely rare. Clinically patient presented with bowel obstruction and associated lower abdominal pain.
- The ultrasonography reported a mass lesion in the intra-abdomen. Thus, a biopsy is taken from the mass lesion that was diagnosed as FA.
- It should be taken into consideration that FA can be raised from EBT in a male patient in the milk line.

Polymastia is a term for two or more than two breasts or ectopic breast tissue (EBT), which is a developmental anomaly and a rare entity^1^. EBT tissue is rare in occurrence; it occurs around 0.4–6% at all. Mostly they are appearing at the embryonic mammary ridge (milk line). The embryonic mammary ridge is extending from the axilla to the groin; it mostly occurs without a nipple and areola, and although commonly asymptomatic, it may manifest symptoms during pregnancy, menstruation, or lactation due to hormonal changes^1^,^2^. Most commonly, EBT can be seen in the axilla; however, it has also been reported in the valva, chest, hip, shoulder, neck, thigh, perineum, upper extremities, and middle back, including the facial region^1^. The EBT is a soft, slow-growing lump. They can make palpable or nonpalpable lumps^2^. Fine-needle aspiration cytology and ultrasonography can easily diagnose the EBT. Like normal breast parenchyma, EBT can also go under the influence of hormonal changes; and may present breast proliferative and nonproliferative changes^1^. EBT presenting as fibroadenoma (FA) is also reported^2^. The most common benign pathology in young females is FA, which usually appears as a well-circumscribed and painless mass. It accounts for around 50% of all benign tumors in first and second decade of life. FA more than greater than 5 cm in size and greater than 500 g weight is called Giant FA, they account 2–4% of FA^3^,^4^. Around 15% of FAs have been reported in the male breast^5^. EBT presenting with carcinoma can also be reported followed by inflammatory changes and FA^6^. Development of pathologies from EBT in female as well as male are rarely documented in literatures^7^. We present an extremely rare case of a 19-year-old male presenting with a mass between the anterior abdominal wall and postcecum, causing obstruction of small bowel at the level of the terminal ileum, and diagnosed with FA arising from EBT.

## Case presentation

**Figure 1:**
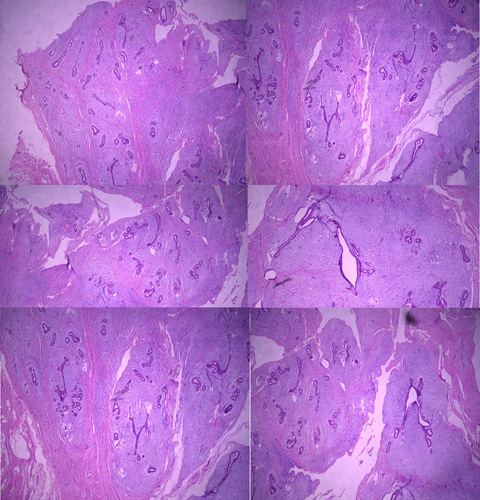
Histopathological examination shows an encapsulated tumor delimited from the surrounding soft tissue, and proliferation of both glandular and stromal elements is observed, which is compatible with fibroadenoma (hematoxylin and eosin staining).

A 19-year-old male referred to Al-Hayat Hospital presented with lower abdominal pain and discomfort for a year with repeated bowel obstruction reported in clinical data; clinically suspicious for tuberculosis and mesenteric lymphadenopathy. On physical examination, there was lower abdominal tenderness. The ultrasonography is reports descending testis, right side hydrocele, ascites, mild splenomegaly, and a few enlarged para-aortic lymph nodes with thick gut loops like structure a computed tomography (CT) scan is suggested. Meanwhile, the peritoneal tab is done and the fluid is sent to the Department of Cytopathology Human Medical Laboratory. Cytology was reported as negative for malignancy and mixed acute and chronic inflammation only. A CT of the abdomen and pelvis with contrast is done; the CT scan suggests a subacute small bowel obstruction due to an underlying infective stricture in the distal ileal loops. The patient gone under laparoscopic surgery congenital band released and biopsy is taken for histopathology examination. Gross examination revealed three pale-brown tissue fragments, the largest of which measures 4×3.5×2.2 cm. The external surface was irregular and lobulated. On cut sectioning, it revealed a gray-white cut surface. No calcification or necrosis was noted. The microscopic examination showed benign parenchymal tissue of the breast that is well-defined, consisting of large, compact mammary glands surrounded by ductal epithelium and myoepithelial cells covered with hyperplastic and fibrotic stroma with no evidence of granuloma or malignancy. The histopathological report was ‘benign breast parenchyma showing fibroadenoma’. The patient was healthy with no evidence of local recurrence after 1 year of follow-up (Fig. 1).

## Discussion

Various rare studies have been published from Afghanistan^8^–^12^. Around 0.4–6% of human beings suffer from EBT, and the incidence is different according to race, sex, and genetics. It is more common in females than in males (5.19 vs. 1.68); accordingly, it has the lowest incidence in white people; however, it has the highest incidence in Japanese. The axilla followed by vulva is the most common location^2^. Around 67% of EBT appear in the thoracic and abdominal regions along the milk line, commonly on the left side of the body. Twenty and 13% appear in the axilla and anywhere in the milk line, respectively. ‘Mammae erraticae’ is a term for EBT in an uncommon location. ETB in uncommon locations is reported in the flank, upper arm, chest, midline of the back, buttocks, back of the neck, and hips^6^. EBT presenting as FA is also reported in face, posterior neck, chest middle back, buttocks, vulvar, and thighs. In this case we present a EBT presented as a FA in intra-abdomen of a young male patient causing intestinal obstruction^13^. FA in the male breast is rare; however, benign breast parenchyma showing FA in the intra-abdomen of a male patient is extremely rare^5^ Sometimes FA of the male breast is considered a nodular foci of gynecomastia^14^. FA is a true case of proliferative breast disease in a male patient. Around 15% of male breast cancer is reported in the literature to date. Hormonal imbalance is one of the causes of proliferative breast diseases such as fibroepithelial lesions or gynecomastia. FA has both estrogen and progesterone receptors; most male breast FAs are reported in patients receiving estrogen therapy for a medical condition like prostate carcinoma or in patients who are male-to-female transgender. Thus, FAs with normal hormonal levels in males are extremely rare; four cases are reported as an idiopathic male breast FA^3^,^5^. There are two hypotheses for EBT in uncommon locations: either during chest development there is a migratory arrest of breast primordium or this can appear from modified apocrine sweat glands. Mostly the EBT is without sign or symptom, except in pregnancy, lactation, and menstruation, when it will appear as pain and swelling of the region^6^. Due to the delay in diagnosis of carcinoma arising from an EBT, they have a poor prognosis. The delay is due to a broad differential diagnosis, including lipoma, vascular neoplasm, suppurative hidradenitis, lymphadenopathy, sebaceous cysts, tuberculosis, and malignancy. Some kidney and urinary tract malformations found to be associated with EBT include adult dominant polycystic kidney disease, cystic renal dysplasia, unilateral renal agenesis, familial renal cysts, and congenital stenosis of the pyeloureteral joint^15^. A suspicious lesion found anywhere in the abdomen and thoracic cavity; FA should also be included in the differential diagnosis. Further evaluation with ultrasound, CT scan, and excisional biopsy should be done to rule out carcinoma in EBT.

This case report is compliant with the SCARE Guidelines 2020^16^.

## Conclusion

When a tumor is palpated in the milk line, the existence of FA should be taken into consideration. FA should also be considered as one of the differential diagnoses, whenever there is intra-abdominal lymphadenopathy or any suspicious lesions, such as tuberculosis. FA of the male EBT in the intra-abdomen is extremely rare. However, a close follow-up of the patient is strongly recommended, as the carcinoma arising from FA has a very poor prognosis.

## Ethical approval

As the case report is a compilation of information from a retrospective period, we obtained exemption from ethical approval from the Institutional Ethical Committee.

## Patient consent

Written informed consent was obtained from the patient for the publication of this case report and accompanying images. A copy of the written consent is available for review by the Editor-in-Chief of this journal on request.

## Sources of funding

None funding.

## Author contribution

Z.B.: patient care and clinical management and manuscript writing. Y.A., J.A., F.R., N.M., S.H.M.: manuscript writing.

## Conflicts of interest disclosure

There are no conflicts of interest.

## Research registration unique identifying number (UIN)

1. Name of the registry: NA.
2. Unique identifying number or registration ID: NA.
3. Hyperlink to your specific registration (must be publicly accessible and will be checked): NA.

## Guarantor

Dr Sayed Hamid Mousavi and Jawad Amini.

## Provenance and peer review

Not commissioned, externally peer-reviewed.

## Associate Professor and Unit Chief

Zekrullah Baset, Cytopathologist, Human Medical Laboratories, Kabul, Afghanistan

## Appendix A. Supplementary data

NA.

## References

[1] Erbaba H Pinar G . Fibroadenoma of bilateral axillary ectopic breast tissue: a rare case report based on Orem’s Self Care theory. Arch Nurs Pract Care 2019;5:8–14.

[2] Teixeira IP Azzopardi C Chowdhury R . Ectopic breast tissue presenting as an enlarging abdominal mass. Radiol Case Rep 2020;15:733–740. 32300469 10.1016/j.radcr.2020.02.028 PMC7152596

[3] Agarwal P Kohli G . Fibroadenoma in the male breast: truth or myth? Turk J Surg 2016;32:208. 10.5152/UCD.2015.3120 PMC4970781 27528814

[4] Yilmaza E Afsarlara ÇE Yilmaz S . Fibroadenoma of ectopic breast tissue of the axilla in an adolescent girl: report of a rare entity. Ann Pediat Surg 2017;13:99–100.

[5] Morikawa H Nobuoka M Amitani M . Fibroadenoma in a young male breast: a case report and review of the literature. Clin Case Rep 2021;9:e05114. 34840799 10.1002/ccr3.5114 PMC8605269

[6] Balmiki P Mourya K . Fibroadenoma of ectopic breast tissue in chest wall: review of the literature and report of a rare case. Highlights Med Med Sci 2021;14:56–61.

[7] Kavesh M Drew P Stewart B . Pathology, immunology, and laboratory medicine, University of Florida, Gainesville, Florida, UNITED STATES|. 2020;7–8.

[8] Daifoladi AA Talemi HG Rezaei MA . Concomitant trans-sternal repair of Morgagni hernia and ventricular septal defect in a patient with Down syndrome: a case report. Int J Surg Case Rep 2022;92:106911. 35245851 10.1016/j.ijscr.2022.106911 PMC8892097

[9] Haghjoo S Mousavi SH Farsi Y . Post-surgery cholesteatoma complicated by facial nerve paralysis: a case report from Afghanistan. Int J Surg Case Rep 2021;82:105916. 33957403 10.1016/j.ijscr.2021.105916 PMC8113744

[10] Mousavi SH Haghjoo S Tahvildari A . Complete laceration of motor branches of facial nerve and its successful repair: a case report from Afghanistan. Int J Surg Case Rep 2021;81:105839. 33887853 10.1016/j.ijscr.2021.105839 PMC8044676

[11] Rahimi MT Shirzad MA Amanat A . Successfully separated conjoined twins at French Medical Institute for Mothers and Children Kabul Afghanistan. Int J Surg Case Rep 2022;92:106886. 35248881 10.1016/j.ijscr.2022.106886 PMC8898893

[12] Zahid SU Taeeb AA Shah J . A parasagittal sinus meningioma in young female adult in Afghanistan. Interdiscip Neurosurg 2021;24:101121.

[13] Lee D . Accessory breast tissue presenting as an anterior abdominal wall mass. Clin Surg 2020;5:2794.

[14] Holleb A Freeman H Farrow J . Cancer of male breast. II. N Y State J Med 1968;68:656–663. 4171099

[15] Amir M Aravind K Shaikh H . A rare case of fibroadenoma in ectopic breast tissue of axilla: case report. Int Surg J 2018;5:3446–3449.

[16] Agha RA Franchi T Sohrabi C . The SCARE 2020 guideline: updating consensus surgical CAse REport (SCARE) guidelines. Int J Surg 2020;84:226–230. 33181358 10.1016/j.ijsu.2020.10.034

